# Supplementary material for: Chromothripsis during telomere crisis is independent of NHEJ, and consistent with a replicative origin
Source: Genome Res. 2019 May;29(5):737–49. doi: 10.1101/gr.240705.118 (PMC6499312; doi:10.1101/gr.240705.118)
Supplement: Supplemental Material [file supp_gr.240705.118_Supplemental_file_1.zip › contigs/annotated_contigs/DB91/contig.2.DB91_length_567_mean_cov_7.93650793651.docx]

**DB91_length_567_mean_cov_7.93650793651**

```
GAATTCCCACATGTTGTGGGAGGAACCTGGTGGGAGGTAATTGAATCATGGGGCAGGTCTTTCCTATGCTGTTCTCATGATAGTGAGTA
 >chr7:115646839-115647140 + E=2e-169
AGTCTCATGAGATCTGATGGTATCATAAGGGAGAGTTTCCCTGCACAATCTCTCTTCTCTTGTCTGCCACCATGTGAGATGTGTCTTTC

ACCTTCCACCATGATTGTGAGGCCTCCCCAGCCATGCGGAACTGTAAGTCGATTAAACCTCTTTCTTTTGTAAATTTCCCAGTGTTGGG

TGTGTAATTACCAGCAGCATGAGAACGGGC|CAAT|GGCATCAATGTCATCAAACCCTCCACTGAGTTCTAAGTGTCTTTTTATAAATC
                               >chr7:115662011-115662281 - E=8e-151
TCCAAAAATATGGTGAGCCCATTCCCTTATCTCTCCCTTGTTAGAGGACACATGTCTCAGACCTCTAACCAGGACATGTCAAGAGGTGG

GCACGCGGGTACTTCCACAGTGCTTTTCTCCTGGCCACCCCTTCCCCTTGTGCCTTTCATAGCTCTCAGGCAGGGCCGATAAGCCTGTT

CTCCCCAGTGCTGAATCTGAGGACCAACTCTGGGG
```
